# Plant-Derived Exosome-like Nanovesicles for Metabolic Dysfunction-Associated Steatotic Liver Disease

**DOI:** 10.3390/vetsci13090851

**Published:** 2026-08-22

**Authors:** Tinghong Kuang, Lijun Wang, Yaning Shi, Ji Cheng, Shifeng Pan

**Affiliations:** 1 College of Veterinary Medicine, Yangzhou University, Yangzhou 225009, China; 18351990713@163.com (T.K.); wlj6379@163.com (L.W.); syn990123@163.com (Y.S.); 2 Jiangsu Co-Innovation Center for Prevention and Control of Important Animal Infectious Diseases and Zoonoses, Yangzhou University, Yangzhou 225009, China

**Keywords:** metabolic dysfunction-associated steatotic liver disease, plant-derived exosome-like nanovesicles, gut microbiota, gut–liver axis, lipid metabolism

## Abstract

Metabolic dysfunction-associated steatotic liver disease (MASLD) is one of the most prevalent chronic liver diseases worldwide, involving multiple pathological mechanisms such as lipid metabolism disorders, oxidative stress, chronic inflammation, insulin resistance, and dysregulation of the gut–liver axis. Currently, there remains a lack of effective and long-term safe therapeutic strategies for MASLD, necessitating the development of novel interventions. Plant-derived exosome-like nanovesicles (PELNs) represent naturally secreted nanoscale vesicles derived from plant cells. Their advantages, including extensive availability, high biocompatibility, low immune activation, remarkable stability, and convenient oral administration, make them attractive platforms for therapeutic delivery. PELNs can not only encapsulate natural bioactive compounds such as polyphenols, alkaloids, terpenoids, and polysaccharides but also serve as drug delivery vehicles to achieve liver-targeted transport and synergistic therapy. Current evidence for PELNs in MASLD is predominantly preclinical. In vitro studies have provided mechanistic insights into their effects on lipid metabolism, oxidative stress, inflammatory signaling, and hepatocellular injury, whereas animal studies have demonstrated improvements in hepatic steatosis and related metabolic abnormalities for selected PELNs. A smaller number of studies have investigated tissue distribution, oral uptake, or preliminary biosafety. However, systematic pharmacokinetic, long-term toxicological, and target-species clinical evidence remains insufficient. Accordingly, the therapeutic effects discussed in this review should be interpreted primarily as preclinical findings, and the applicability of PELNs in veterinary practice remains to be established.

## Simple Summary

Metabolic dysfunction-associated steatotic liver disease (MASLD), a metabolic disorder affecting millions of individuals worldwide, is characterized by excessive hepatic lipid deposition accompanied by oxidative imbalance, persistent inflammation, impaired insulin signaling, and disruption of gut–liver axis regulation interactions. Although several therapeutic approaches have been developed, current interventions remain insufficient for achieving sustained disease control, emphasizing the importance of exploring alternative treatment strategies. Plant-derived exosome-like nanovesicles (PELNs) are emerging as naturally derived nanostructures with considerable potential in biomedical applications. These vesicles possess favorable features, including good physiological compatibility, minimal immune stimulation, structural stability, and the possibility of oral administration. In addition to acting as carriers for functional molecules, PELNs naturally contain diverse plant metabolites, such as phenolic compounds, alkaloids, terpenoids, and polysaccharides, which may contribute to their therapeutic activities. Current preclinical evidence indicates that selected PELNs can reduce hepatic lipid accumulation and liver injury in experimental models of MASLD/NAFLD or hepatic steatosis. In addition, studies conducted in obesity, diabetes, insulin resistance, gut dysbiosis, and other metabolically related models provide indirect evidence relevant to MASLD pathophysiology. These indirect findings support potential metabolic, inflammatory, and gut–liver regulatory effects of PELNs. This review provides an overview of the properties, preparation approaches, and therapeutic applications of PELNs in MASLD. In addition, current limitations and future opportunities for their clinical development are discussed to facilitate the translation of PELNs into innovative and naturally based therapies for metabolic liver diseases.

## 1. Introduction

Metabolic dysfunction-associated steatotic liver disease (MASLD), formerly known as non-alcoholic fatty liver disease (NAFLD), was renamed under the new nomenclature to better reflect the metabolic dysfunction underlying the disease. In this review, the term MASLD is used consistently. It is characterized by fat accumulation in hepatocytes accounting for at least 5% or more of liver weight, accompanied by metabolic abnormalities such as overweight or obesity and insulin resistance [1]. MASLD may progress to metabolic dysfunction-associated steatohepatitis (MASH), characterized by inflammation and cellular damage, ultimately leading to liver fibrosis and hepatocellular carcinoma, with the former indicating a higher risk of mortality in patients [2]. In animal farming, fatty liver can occur in various livestock species, affecting not only animal health but also reducing farming efficiency and causing significant economic losses. The treatment options for MASLD are diverse and primarily divided into conservative therapy and surgical therapy [3]. Although improving dietary patterns and increasing exercise intensity yield favorable outcomes, significant non-compliance remains, necessitating more suitable treatment strategies.

This review aims to summarize relevant preclinical evidence demonstrating that plant-derived exosome-like nanovesicles (PELNs) are emerging as a promising therapeutic agent for MASLD/MASH. Extracellular vesicles (EVs) are spherical particles enclosed by a lipid bilayer membrane, enriched with various bioactive molecules such as proteins, lipids, and nucleic acids [4,5]. EVs are naturally secreted from a wide range of cellular sources and extensively exist in circulating plasma and other physiological fluids, exhibiting excellent biosafety, biocompatibility, and structural stability [6,7,8]. Compared to other drugs, EVs possess unique multifunctionality and targeting capabilities, demonstrating significant therapeutic potential in the treatment of diseases such as diabetes, cancer, and cardiovascular disorders [9,10,11]. However, extracellular vesicles derived from animals have low yields, high production costs, and may also cause harm to organisms [12,13,14]. Conventional plant extracts provide abundant bioactive compounds, but these components are generally present as free or non-vesicle-associated molecules and may be limited by instability, degradation, and variable bioavailability after administration. In contrast, PELNs combine plant-derived bioactive cargo with a lipid-bilayer vesicular structure, which may protect encapsulated molecules and facilitate their delivery to recipient cells. Therefore, PELNs are distinct from conventional plant extracts because their biological effects may arise not only from plant-derived active compounds but also from vesicle-mediated protection and delivery. Compared with mammalian cell-derived EVs, PELNs offer advantages such as low cost, high stability, broad availability of plant raw materials, and potential suitability for oral administration in animals. Additionally, they can be used to load and deliver exogenous active substances and drugs [15,16,17,18,19,20]. Preclinical studies have reported anti-inflammatory, immunomodulatory, and drug-delivery activities of PELNs in cellular and animal models of several diseases [21,22,23,24]. However, these findings should not be interpreted as evidence of established therapeutic efficacy in veterinary practice. The current literature therefore primarily supports the preclinical potential of PELNs and provides a rationale for further pharmacokinetic, toxicological, and target-species investigations. This article reviews research data with promising clinical potential, demonstrating that PELNs possess broad sources, diverse targets, and high bioavailability. In preclinical models, selected PELNs have been reported to alleviate hepatic injury and regulate metabolic pathways relevant to MASLD, including lipid metabolism and the gut–liver axis. However, the generalizability of these findings to livestock metabolic liver disease remains to be established. Importantly, it remains unclear whether the biological advantages of PELNs over conventional plant preparations arise primarily from their vesicular structure, their encapsulated bioactive cargos, or the combined effects of both. It also remains uncertain whether PELNs consistently provide greater stability, bioavailability, tissue delivery, or biological efficacy than corresponding plant extracts, and whether their proposed advantages over mammalian EVs can be maintained after purification, standardization, and large-scale production. These unresolved questions represent an important knowledge gap in the current PELN field. Therefore, this review critically evaluates the available preclinical evidence for PELNs in MASLD, with particular attention to their distinctive features, mechanistic basis, evidence strength, and the major limitations that currently restrict reproducibility and future veterinary translation.

This article was conducted as a narrative review with a structured and transparent literature-search strategy. Through the search of databases such as PubMed, Web of Science, and Scopus, relevant studies were identified. No strict publication-date restriction was initially applied; however, priority was given to studies published during the past 10 years. Earlier landmark studies were included when necessary. Search terms included combinations of “plant-derived exosome-like nanovesicles”, “PELNs”, “MASLD”, “MASH”, “fatty liver”, “lipid metabolism”, “gut microbiota”, and “gut–liver axis”. Studies were included if they focused on plant-derived vesicles or provided mechanistic evidence relevant to MASLD. Priority was given to original PELN studies in cellular or animal models. Studies on isolated plant bioactive compounds were included only as indirect mechanistic evidence, whereas articles unrelated to PELNs or MASLD were excluded.

## 2. Metabolic Dysfunction-Associated Steatotic Liver Disease (MASLD)

### 2.1. Concept of MASLD

Metabolic dysfunction-associated steatotic liver disease (MASLD), previously known as NAFLD, is a term representing the hepatic manifestation of a multisystem disease [25]. The former is defined as ≥5% hepatic steatosis and at least one cardiovascular metabolic risk factor such as dyslipidemia or obesity, with no other underlying causes and minimal or no alcohol consumption [26]. MASLD is currently considered one of the most prevalent chronic liver diseases worldwide, affecting approximately 25–30% of the adult population and being more prevalent in individuals with obesity and type 2 diabetes, closely associated with metabolic syndrome [27]. It is an umbrella term for a group of liver diseases, including fatty liver disease and metabolic dysfunction-associated steatohepatitis (MASH), which in severe cases are capable of advancing to cirrhosis and hepatocellular carcinoma [28,29]. Hepatic steatosis is a hallmark feature of the disease, resulting from excessive nutrition and a monotonous diet rich in glucose, high-fructose, and saturated fats, which lead to continuous accumulation of triglycerides in the liver through multiple pathways [30,31]. MASH is a progressive liver disease that can lead to various liver-related complications and significantly increase patient mortality [32]. Its histological features include steatosis, lobular inflammation, and hepatocyte ballooning, with or without fibrosis, and it is an important risk factor for the fastest progression of liver cancer [33,34]. In MASH, impaired immune tolerance mechanisms and upregulated expression of pro-inflammatory cytokines drive excessive extracellular matrix deposition in the liver, frequently progressing to hepatic fibrosis [35,36,37,38]. The pathogenesis of the latter is associated with endothelial cell injury, activation of inflammatory immune cells, and hepatic stellate cells (HSCs) activation [39,40,41].

**Figure 1:**
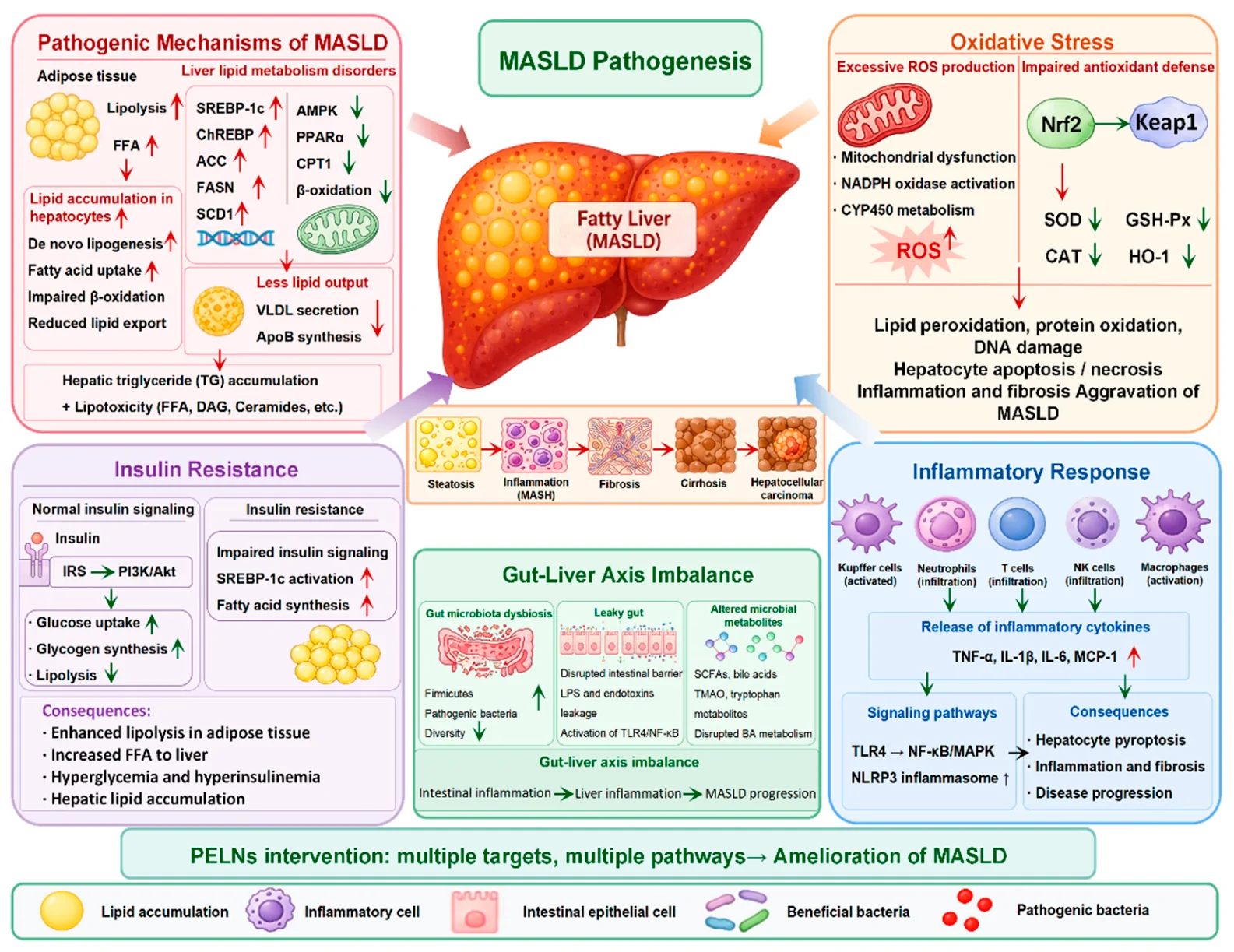
The pathogenesis of metabolic dysfunction-associated steatotic liver disease (MASLD).

The pathogenesis of MASLD is highly heterogeneous, typically resulting from the coordinated activation of multiple pathological pathways, including insulin resistance, dysregulation of glucose and lipid metabolism, organelle stress, inflammatory responses, intestinal-liver axis imbalance, genetic susceptibility, and adipose tissue dysfunction (Figure 1).

### 2.2. Pathogenesis of MASLD

#### 2.2.1. Lipid Metabolism Disorders

Lipid metabolism disorders serve as the pathological basis for the onset and progression of MASLD and represent a critical driver of disease advancement. The liver is a pivotal organ for systemic lipid metabolism. Under physiological conditions, it maintains hepatic lipid homeostasis by orchestrating processes including fatty acid uptake, de novo lipogenesis (DNL), fatty acid β-oxidation, and the assembly and secretion of very low-density lipoprotein (VLDL) [33]. Persistent metabolic stress, including obesity, insulin resistance, and high-fat or high-sugar diets, disrupts hepatic lipid homeostasis, resulting in increased lipid uptake and synthesis, reduced fatty acid oxidation and export, and ultimately hepatic triglyceride accumulation and steatosis.

In MASLD, hepatic fatty acids arise mainly from adipose tissue lipolysis, dietary intake, and de novo lipogenesis, with adipose-derived FFAs contributing approximately 60% of the hepatic fatty acid pool [1]. Secondly, in a high-sugar and high-insulin environment, sterol regulatory element-binding protein-1c (SREBP-1c) and carbohydrate response element-binding protein (ChREBP) are continuously activated, inducing an increase in the expression of lipid synthesis-related enzymes such as acetyl-CoA carboxylase (ACC), fatty acid synthase (FASN), and stearoyl-CoA desaturase 1 (SCD1) [37]. Recent studies have shown that AMP-activated protein kinase (AMPK), as an important sensor of cellular energy metabolism, can negatively regulate fat production by inhibiting ACC activity and downregulating the expression of SREBP-1c [1]. In MASLD, reduced AMPK activity promotes lipogenesis, while impaired peroxisome proliferator-activated receptor alpha (PPARα) signaling limits fatty acid β-oxidation through enzymes such as carnitine palmitoyltransferase 1 (CPT1) and acyl-CoA oxidase 1 (ACOX1) [42]. Reduced PPARα activity in MASLD impairs fatty acid oxidation, promoting triglyceride accumulation and lipotoxicity, which is considered a major driver of disease progression [29]. Although triglycerides serve to some extent as a protective form for storing excess fatty acids, the persistent accumulation of lipotoxic lipids such as FFAs, ceramides, diacylglycerols, and free cholesterol can promote hepatocyte apoptosis and necrosis, ultimately driving the progression of MASLD to MASH and liver fibrosis.

Overall, disrupted hepatic lipid homeostasis promotes lipid accumulation and lipotoxicity, making the restoration of lipid balance a key therapeutic target in MASLD.

#### 2.2.2. Oxidative Stress

Under normal physiological conditions, a dynamic balance is maintained between the generation of reactive oxygen species (ROS) and the antioxidant defense system within the body, with small amounts of ROS participating in physiological processes such as cell signaling, energy metabolism, and immune regulation. However, in MASLD patients, abnormal lipid accumulation in hepatocytes and persistent buildup of lipotoxic substances lead to significantly increased ROS production, coupled with reduced antioxidant capacity, resulting in oxidative stress imbalance [32]. Oxidative stress induces lipid peroxidation, protein and DNA damage, thereby promoting the progression from steatosis to MASH and fibrosis. Mitochondrial dysfunction is a major source of ROS, as elevated FFAs enhance mitochondrial β-oxidation and increase oxidative burden [43]. However, prolonged excessive fatty acid oxidation increases the load on the electron transport chain, triggering electron leakage and the generation of large amounts of ROS, which further damage mitochondrial DNA, respiratory chain complexes, and mitochondrial membrane structures, reduce ATP synthesis efficiency, creating a vicious cycle that exacerbates hepatocyte injury [37]. In addition to mitochondria, endoplasmic reticulum stress, peroxisomes, and the cytochrome P450 enzyme system are also important sources of ROS in MASLD [44]. Excessive ROS promote lipid peroxidation and damage proteins, DNA, and cell membranes, ultimately inducing hepatocyte apoptosis or necrosis [45]. Moreover, lipid peroxidation products can act as damage-associated molecular patterns (DAMPs), activating Kupffer cells and other immune cells, promoting the release of inflammatory mediators, thereby further exacerbating liver tissue injury [38].

Recent studies have shown that oxidative stress is closely associated with inflammatory responses, and the two mutually promote each other, jointly driving the progression of MASLD. ROS can activate the NF-κB signaling pathway, facilitating the synthesis of pro-inflammatory cytokines such as TNF-α, IL-1β, and IL-6 [18]. In addition, ROS serve as a critical inducer of the activation of the NOD-like receptor pyrin domain-containing protein 3 (NLRP3) inflammasome. NLRP3 inflammasome activation promotes caspase-1-dependent release of IL-1β and IL-18 and hepatocyte pyroptosis, thereby aggravating MASH and fibrosis [46]. Under physiological conditions, nuclear factor erythroid 2-related factor 2 (Nrf2) is bound to the Keap1 protein and remains inactive [47]. Under oxidative stress conditions, Nrf2 undergoes dissociation from Keap1 prior to its translocation into the nucleus, inducing the expression of various antioxidant enzymes such as heme oxygenase-1 (HO-1), superoxide dismutase (SOD), and glutathione peroxidase (GPx), thereby eliminating ROS and alleviating oxidative damage [45]. Persistent ROS accumulation in MASLD can impair Nrf2-mediated antioxidant defenses, further aggravating oxidative stress. Overall, oxidative stress contributes to MASLD progression through mitochondrial dysfunction, lipid peroxidation, inflammation, and hepatocellular injury, making antioxidant pathways such as Nrf2 an important target for PELN-related intervention.

#### 2.2.3. Inflammatory Response

In recent years, the mechanism of MASLD has gradually evolved from the traditional “two-hit hypothesis” to the “multiple-hit hypothesis”. It is believed that multiple pathological factors such as lipid metabolism disorders, oxidative stress, imbalance of intestinal flora, and genetic factors jointly induce chronic liver inflammation, ultimately leading to liver cell damage, activation of hepatic stellate cells, and liver fibrosis [40]. When liver cells are damaged, DAMPs such as mitochondrial DNA, high mobility group protein B1 (HMGB1), and ATP are released and recognized by the innate immune cells of the liver, thereby activating the innate immune response and promoting the continuous release of inflammatory mediators [38]. Kupffer cells (KCs) are the resident macrophages in the liver. During the homeostasis stage, KCs remove metabolic substances from the body and maintain a tolerant immune environment within the liver. When the liver is damaged, KCs are activated, releasing a large amount of inflammatory cytokines and chemokines, and initiating inflammatory signals through pattern recognition receptors such as Toll-like receptors (TLRs) [48]. TLR4 activation triggers MyD88-dependent NF-κB and mitogen-activated protein kinase (MAPK) signaling, promoting pro-inflammatory cytokine production and hepatic inflammation; therefore, suppression of these inflammatory pathways represents a potential target for PELN-related intervention [48]. In addition to innate immunity, the inflammatory response also involves interactions among various immune cells. During the progression of MASLD, circulating monocytes, neutrophils, natural killer cells (NK cells), and T lymphocytes continuously infiltrate the liver tissue, jointly participating in the formation of the inflammatory microenvironment [41]. Furthermore, due to the mutual promotion of the mechanisms of inflammatory response and oxidative stress, when there is excessive ROS and mitochondrial dysfunction, the NLRP3 inflammasome is activated, leading to hepatocyte pyroptosis [46]. During this process, a substantial quantity of inflammatory mediators is released, forming a persistent inflammatory amplification cycle that exacerbates MASH and liver fibrosis and drives progression toward hepatocellular carcinoma.

#### 2.2.4. Insulin Resistance

Insulin resistance (IR) is often associated with obesity, type 2 diabetes and metabolic syndrome, and leads to liver fat deposition. Under normal physiological conditions, insulin binds to IR, activating insulin receptor substrates (IRS) and their downstream phosphatidylinositol 3-kinase (PI3K)/protein kinase B (Akt) signaling pathways, promoting glucose uptake and glycogen synthesis, while inhibiting lipolysis in adipose tissue and gluconeogenesis in the liver, thereby maintaining the homeostasis of glucose and lipid metabolism [49]. Obesity, high-calorie diets, and chronic inflammation impair insulin signaling, reducing insulin sensitivity and disrupting glucose and lipid homeostasis, thereby promoting hepatic lipid accumulation [50]. After IR occurs, lipolysis in adipose tissue is enhanced, leading to the release of large amounts of FFA into circulation and their transport to the liver, thereby increasing hepatic lipid load. Meanwhile, excessive FFAs not only promote triglyceride synthesis but also generate lipotoxic metabolites such as ceramides and diacylglycerols, further exacerbating hepatocyte damage [51].

On the other hand, since IR is accompanied by high insulinemia, the liver glucose production (HGP) is not inhibited by insulin, resulting in increased expression of the rate-limiting enzymes for glucose synthesis (PEPCK) and glucose-6-phosphatase (G6Pase), and continuous elevation of hepatic glucose output, leading to a state of hyperglycemia [52]. Despite insulin resistance, hepatic SREBP-1c signaling may remain active, increasing ACC and FASN expression and thereby promoting de novo lipogenesis [53]. Pro-inflammatory cytokines such as TNF-α and IL-6 activate JNK and NF-κB signaling, promoting inhibitory IRS-1 phosphorylation and further impairing insulin signaling [54,55]. In summary, IR not only promotes the release of FFAs from adipose tissue and increases lipid accumulation in the liver, but also contributes to the development of MASLD through multiple mechanisms, including suppression of the PI3K/Akt signaling pathway, enhanced de novo lipogenesis, and induction of inflammation and oxidative stress.

#### 2.2.5. Gut–Liver Axis Imbalance

The gut microbiota is not only a classified biological ecosystem, but also participates in various functions of the body’s intestines and beyond, such as immune system regulation, nutrient absorption, digestion, and lipid metabolism [56]. The intestine is connected to the liver through the enterohepatic axis, and the intestinal microbiota serves as the link between these two organs [57]. Relevant clinical studies have shown that there are marked variations in the constitution of intestinal microorganisms between obese individuals and non-obese individuals [58]. The changes in the structure of the intestinal microbial community often promote the occurrence of inflammation and liver fibrosis [59]. Therefore, the intestinal microbial community is of great significance in the pathogenesis of MASLD. The gut–liver axis refers to the anatomical and functional relationship between the gastrointestinal tract and the liver [60]. Changes in this axis can lead to alterations in intestinal permeability, thereby facilitating the growth of pathogenic bacteria.

In MASLD, there is significant dysbiosis of the gut microbiota, which presents as a dominance of *Firmicutes* and a marked reduction in α-diversity [61,62]. The cross-signaling between the intestine and the liver is transmitted through the intestinal barrier. A complete intestinal barrier can restrict the migration of bacterial products while allowing nutrients to pass through [63]. The disruption of the intestinal barrier is known as “leaky gut” or intestinal permeability syndrome, characterized by the release of bacterial metabolites and endotoxins into the circulatory system [64]. This condition is associated with the development of various metabolic and autoimmune disorders, including obesity and MASLD. Organelle dysfunction, particularly in mitochondria, can increase ROS production and further aggravate oxidative stress in MASLD [45]. The excessive expression of related genes caused by oxidative stress can lead to the disruption of intestinal barrier integrity, thereby resulting in microbial imbalance and bacterial invasion [65]. The imbalance of microbial metabolites, including short-chain fatty acids (SCFAs), bile acids (BAs), endogenous ethanol, tryptophan metabolites, trimethylamine and branched-chain amino acids, has been shown to contribute to the development of MASLD and HCC [66]. Primary bile acids contribute to gut–liver homeostasis by regulating intestinal barrier function, glucose and lipid metabolism, and inflammatory responses [67]. Secondary bile acids, such as deoxycholic acid, can antagonize farnesoid X receptor (FXR) signaling and thereby promote bile acid synthesis [68]. Gut microbiota dysbiosis and intestinal barrier dysfunction can aggravate liver injury and disease progression [69]. The intestinal microbiome characteristics of decompensated liver cirrhosis are characterized by an increase in the relative abundance of potential pathogenic bacteria, while the abundance of symbiotic bacterial communities with potential probiotic effects decreases, especially in *Lactobacillaceae* and *Ruminococcaceae* [70,71,72].

The above studies indicate that the gut microbiota in MASLD patients is significantly altered and exacerbates a series of systemic inflammatory responses through negative feedback. However, leveraging this regulatory relationship, numerous clinical trials have demonstrated that fecal microbiota transplantation (FMT) can be used to treat MASLD [73,74,75,76]. Currently, traditional approaches such as probiotics, prebiotics, synbiotics, and postbiotics, along with emerging methods including engineered probiotics or phage therapy, are all considered promising therapeutic strategies [77,78]. However, the former exert positive effects only under healthy conditions, while the latter still lack clinical maturity [79]. However, FMT may cause adverse reactions such as diarrhea, abdominal pain, bloating, and nausea, which are usually mild and self-limiting and may be related to the immune stress response it induces or the underlying disease itself [80].

### 2.3. Current Therapeutic Approaches for MASLD

Cirrhosis is the end-stage of fibrosis, and if left untreated, it may progress to liver cancer [81,82]. Therefore, the severity of liver fibrosis is often considered the most important liver-related factor associated with predicting adverse clinical outcomes and mortality in MASH patients [83,84,85]. Nevertheless, clinically effective antifibrotic therapy remains difficult to achieve, and whole liver transplantation is currently the most effective treatment for end-stage fibrosis [86,87]. To prevent progression to end-stage disease, numerous clinical studies have shown that early lifestyle interventions and bariatric surgery can effectively avoid adverse outcomes [88,89,90]. However, weight reduction and dietary optimization require long-term adherence and intervention. During the course of the disease, patients also need anti-obesity medications as adjunctive therapy, such as orlistat (a pancreatic lipase inhibitor), GLP-1 agonists (semaglutide, liraglutide), GIP/GLP-1 dual receptor agonists (tirzepatide), and statins (atorvastatin, rosuvastatin) [91,92,93]. These drugs can improve liver function indicators such as transaminase levels in some patients, but there is insufficient evidence to prove that they can reverse fatty liver or liver fibrosis [94]. Therefore, they can only be used as an adjunctive treatment. Some studies have also pointed out that the duration of these clinical drug treatments is unknown, and they have many side effects [95]. If the treatment is stopped, patients may experience weight gain and even aggravate the irreversible damage to the liver. Although new drug treatments have emerged, offering promising options, their high cost and scarcity of resources still make them unsuitable as the best choice for patients [96]. Therefore, PELNs, as promising drugs with low cost, abundant availability and low biological toxicity, bring new possibilities for the treatment of MASLD/MASH. In the following section of this review, we will discuss the intervention and therapeutic effects of plant-derived exosome-like nanovesicles on this disease based on its pathogenesis.

## 3. Plant-Derived Exosome-like Nanovesicles (PELNs)

### 3.1. Extracellular Vesicles

Extracellular vesicles (EVs) are membrane-delimited particles released from cells that are unable to replicate independently. According to the MISEV2023 guidelines, “extracellular vesicle” is recommended as the generic term when the precise biogenesis of a vesicular population has not been established [97]. In this review, the term EV is therefore used for extracellularly released lipid-bilayer vesicles. EVs are rich in proteins, lipids, glycocalyx, and other components on their surface, where surface proteins serve not only as biomarkers for identifying cellular origin but also as mediators of various biological functions [98]. EVs can be classified into several subgroups based on their biological characteristics: (i) Exosomes are a subtype of extracellular vesicles secreted by most human cells, with a diameter of approximately 30–150 nm, appearing as cup-shaped, double-membrane structures under transmission electron microscopy; (ii) microvesicles (MVs) are larger vesicles ranging from about 100 to 1000 nm in size, formed by budding and subsequent fission from the cell surface, and contain cellular contents such as genetic material, proteins, and lipids; (iii) apoptotic bodies (ABs) are EVs released by apoptotic cells, measuring approximately 0.5–5 μm in size, formed primarily through membrane blebbing, protrusion of the cell membrane, and subsequent fragmentation, and contain DNA, RNA, proteins, and lipid molecules [99]. EVs can be widely secreted by various cell types and isolated from multiple biological sources, including bodily fluids, tissue samples, and cell culture supernatants [100]. Among the common sources mentioned above, biological fluids have become a research focus due to their clinical ease of sampling and strong potential for dynamic monitoring. Blood is the most commonly used biological fluid for obtaining and studying EVs, and levels of red blood cell-derived extracellular vesicles (RBCEVs) are significantly altered under various pathological conditions, making them important biomarkers for disease diagnosis [101,102,103]. In addition, it is also frequently used to conduct minimally invasive tests using biological fluids such as blood, saliva, and urine, which is known as liquid biopsy [104]. This has become a revolutionary strategy for cancer diagnosis and prognosis prediction. Furthermore, EVs present in urine and saliva have also been shown to contain disease-related proteins, which may serve as potential biomarkers for neurological disorders such as Alzheimer’s disease or Parkinson’s disease [105,106]. As carriers of diverse biomolecules, EVs can target recipient cells and facilitate the transfer of molecular cargo, thereby playing an important role in intercellular communication [107]. The process is roughly as follows: the plasma membrane invaginates to form mature multivesicular bodies (MVBs) [108]. After these MVBs fuse with lysosomes, the contents of the lysosomes are degraded, or they fuse with the plasma membrane and release exosomes into the extracellular space. In addition, EVs possess a relatively stable membrane-bound structure that is protected by extracellular enzymes, preventing degradation and enabling them to maintain biological stability for a long period of time [109]. Therefore, EVs can also be used in the treatment of many diseases, such as rheumatoid arthritis, diabetes, and cardiovascular diseases [108,110,111]. Apart from biological fluids and cell culture supernatants, milk, plants, and bacteria are also widely used as important sources of EVs [112,113,114]. These new sources have advantages such as lower costs, higher output, and the ability to be scaled up for production, providing a feasible path for the large-scale production of EVs [115]. Given these advantages and considering the need for large-scale production and the multi-faceted medicinal value required for different diseases, the present review concentrates on plant-derived exosome-like nanovesicles.

### 3.2. PELNs

#### 3.2.1. Terminology and General Features of PELNs

Plant-derived exosome-like nanovesicles (PELNs) are heterogeneous nanoscale, membrane-delimited vesicular preparations obtained from plant materials. Although many of these vesicles share certain physicochemical characteristics with mammalian exosomes, including nanoscale size, lipid-bilayer structure, morphology, and molecular cargo, their biogenesis and extracellular origin have not been consistently demonstrated. In mammalian systems, the term “exosome” refers specifically to an extracellular vesicle formed through the endosomal/multivesicular body pathway, and particle size or morphology alone is insufficient for such classification. Therefore, the suffix “-like” is used in the plant literature to indicate phenotypic resemblance to mammalian exosomes without implying an identical biogenesis pathway [97,116]. In this review, PELNs is used as an operational umbrella term for nanoscale membrane-delimited vesicular preparations derived from plant materials and commonly described in the original literature as plant-derived exosome-like nanovesicles, exosome-like nanoparticles, or related terms. In this article, the term “exosomes” is only employed when the existence of an endosomal biosynthetic pathway has been experimentally confirmed. The main components of PELNs include proteins, lipids, nucleic acids and secondary metabolites [117]. PELNs contain proteins derived from multiple cellular and membrane-associated compartments. Several proteins, including PENETRATION 1 (PEN1), PENETRATION 3 (PEN3), and TETRASPANIN 8 (TET8), have been reported as EV-associated proteins in specific plant systems; however, they should not be regarded as universally validated markers for all plant-derived vesicles [116]. At present, no universally accepted molecular marker set has been established for PELNs, and their identification should therefore rely on a combination of physicochemical, morphological, and biochemical characterization rather than on a single protein marker. Lipid components mainly include sphingolipids, phosphatidic acid (PA), sphingomyelin (SM), phosphatidylserine (PS), and cholesterol [118]. These lipids are not only crucial for maintaining the integrity of PELNs membrane structure, but also participate in their biogenesis and extracellular release. The nucleic acids encapsulated in vesicles are mainly RNA and a small amount of DNA, which can mediate intercellular communication and regulate gene expression. In addition, PELNs can accumulate and transport plant-derived secondary metabolites, such as flavonoids, saponins, polysaccharides and alkaloids. These natural active components demonstrate the value for disease intervention [119]. Compared to animal-derived extracellular vesicles, PELNs offer higher production efficiency, shorter extraction cycles, broader availability of raw materials, and lower immunogenicity [115]. Furthermore, plants do not carry pathogens of zoonotic diseases of mammalian origin or human-specific pathogens [120]. Therefore, the nanoparticles derived from plants have excellent biological safety, immunocompatibility and low immunogenicity, providing important security guarantees for their biomedical applications [14]. Due to their outstanding physicochemical properties and biological characteristics, PELNs have attracted extensive attention in recent years, with their potential functions and mechanisms of action being increasingly explored by researchers. Meanwhile, since plants themselves are rich in various natural bioactive compounds, PELNs derived from different plant sources typically retain some of the biological traits and pharmacological activities of their parent plants. As a result, numerous PELNs have been successfully isolated, characterized, and applied in diverse fields such as disease treatment, drug delivery, and functional food development, demonstrating broad application value and promising clinical translation prospects.

#### 3.2.2. Extraction of PELNs

To date, various methods have been used to isolate and purify these PELNs, including ultracentrifugation, density gradient centrifugation, size exclusion chromatography (SEC), polyethylene glycol (PEG) precipitation, and commercial kit-based approaches [100]. The most commonly adopted approach to purify EVs at present is ultracentrifugation, recognized as the “gold standard” for EV isolation and purification due to its relatively simple operation, high yield, and superior purity. This method first removes dead cells and large cellular debris by low-speed centrifugation, followed by ultracentrifugation to sediment vesicles of similar size, thereby achieving their enrichment and initial purification. Due to the presence of numerous impurities such as chloroplast fragments, cell wall debris, and apoptotic bodies in plant sap, centrifugation is often difficult. Therefore, PELNs are often extracted by combining differential centrifugation with sucrose density gradient centrifugation. According to this method, extracellular vesicles typically reside in the intermediate layer between 30% and 45% sucrose solutions [14]. Polymer precipitation methods typically use polyethylene glycol as the medium, collecting exosomes by centrifugation to reduce their solubility [13]. The separation principle of SEC is that high-molecular-weight substances are difficult to penetrate the internal pores of the gel medium, while low-molecular-weight substances can freely enter the gel pores and remain for a longer time, subsequently being eluted later by the mobile phase [121]. Currently, the extraction methods of exosomes have their own advantages, but they also have varying degrees of limitations. The ultracentrifugation method relies on an ultracentrifugation device, and the operation is time-consuming. Moreover, due to the similar sedimentation coefficients of different particles, it is prone to co-precipitate protein aggregates, lipoproteins, and other extracellular vesicles, affecting the purity of the sample. The PEG precipitation method has poor selectivity and is prone to simultaneously precipitating soluble proteins, lipoproteins, and other impurities, resulting in a lower sample purity. At the same time, the residual PEG may interfere with subsequent proteomics, nucleic acid analysis, and functional experiments, thereby affecting the accuracy of the experimental results. The SEC separates based on the hydrodynamic radius of the particles, which can effectively reduce the contamination of soluble proteins and other impurities while maintaining the integrity and biological activity of PELN structure to a large extent. However, the SEC column has certain limitations on the sample loading volume. For low-concentration samples, it usually requires pre-concentration, and the amount of sample that can be processed in a single run is limited, resulting in relatively lower efficiency when processing large-volume samples.

The above-mentioned summary regarding the extraction methods of PELNs is presented in Table 1.

#### 3.2.3. Application of PELNs

Beyond metabolic liver disease, PELNs have also been investigated in other biomedical fields, including tissue repair [122,123,124], inflammatory disorders [125,126], cancer [127], and neurological diseases [128,129]. These studies highlight the broad biological activities and delivery potential of PELNs; however, detailed discussion of these applications is beyond the scope of this review. Here, we focus specifically on their potential roles in MASLD and the mechanisms underlying their therapeutic effects. In this review, we focus on evaluating the application value and clinical translation potential of PELNs in metabolic liver diseases. The occurrence and progression of metabolic liver diseases involve multiple pathophysiological processes, including lipid metabolism disorders, persistent activation of oxidative stress, chronic mild inflammatory responses, and imbalance of the gut–liver axis. Leveraging their inherent bioactive constituents and the delivery advantage of preferential accumulation in the liver via the portal vein following oral administration, PELNs have emerged as a pivotal research direction in the therapeutic landscape of MASLD. PELNs derived from plants such as ginger [21], garlic [125], and blueberries [130] are rich in bioactive compounds including flavonoids, polyphenols, polysaccharides, and terpenoids. With excellent biocompatibility, safety, and oral bioavailability, they can not only serve as functional dietary supplements for the early prevention and intervention of MASLD but also be engineered via nanotechnology to deliver lipid-lowering drugs, enabling targeted liver delivery and synergistic therapy.

### 3.3. Comparison of PELNs with Other Emerging MASLD Interventions

Emerging strategies for MASLD intervention include microbiota-targeted approaches, pharmacological agents, and nanotechnology-based delivery systems, each of which exhibits distinct strengths and limitations. Probiotics provide a relatively simple and non-invasive means of modulating the gut microbiota and may improve intestinal barrier function and microbial metabolite profiles. However, their effects are often strain-dependent and can vary substantially according to host microbiota composition, diet, and disease status [75]. Fecal microbiota transplantation (FMT) enables broader reconstruction of the intestinal microbial community and may therefore exert more extensive effects on the gut–liver axis [73,74,76]. Nevertheless, donor variability, inconsistent microbial engraftment, potential pathogen transmission, and difficulties in standardization remain important barriers to its widespread application.

Small-molecule pharmacotherapies have the advantages of defined chemical composition, reproducible dosing, and relatively well-characterized molecular targets and pharmacokinetic properties [77,131]. However, individual agents generally act on a limited number of pathogenic pathways and may require prolonged administration, while adverse effects, cost, and species-specific differences may constrain their application, particularly in veterinary settings [79]. Synthetic nanoparticles, including lipid- and polymer-based nanocarriers, provide considerable flexibility in particle engineering, surface modification, drug loading, and tissue-targeted delivery. Their clinical translation, however, may be limited by manufacturing complexity, material-associated toxicity, biodegradation concerns, and regulatory requirements [78].

PELNs differ from these approaches because they combine a naturally derived lipid-bilayer vesicular structure with endogenous plant bioactive cargos. This feature may allow PELNs to simultaneously function as biologically active vesicles and as delivery carriers, with potential effects on hepatic metabolism, oxidative stress, inflammation, and gut–liver axis homeostasis. Their plant origin, potential oral applicability, and broad availability may also offer practical advantages for future veterinary use. Nevertheless, these potential benefits should not be interpreted as evidence that PELNs are superior to existing interventions. Compared with conventional drugs and synthetic nanocarriers, PELNs currently have less standardized composition, less predictable pharmacokinetic behavior, substantial batch-to-batch variability, and considerably weaker clinical and target-species evidence. Therefore, PELNs should presently be regarded as a complementary experimental platform rather than a replacement for established pharmacological or microbiota-directed therapies.

## 4. Potential Mechanistic Contributions of PELN-Associated Plant Bioactive Compounds to MASLD

### 4.1. Application Potential of PELNs as Nanocarriers

Although there are already many mature research and development systems and widely used clinical treatment drugs for fatty liver diseases in the market, no single drug has been able to simultaneously address all the issues such as fat deposition, fibrosis, and long-term safety [94]. Although new targeted drugs have significantly better efficacy than traditional liver-protecting drugs, they still have limitations such as limited applicability and high costs. Natural active components derived from plants have attracted attention due to their multiple biological activities. However, most plant active components still have disadvantages such as poor water solubility, insufficient stability, low bioavailability, poor tissue targeting, and rapid metabolism in the body, which limit their clinical application effects. Therefore, in recent years, PELNs, as an emerging natural nanodelivery carrier, have provided new ideas for the treatment of MASLD [114]. PELNs possess several advantages such as excellent biocompatibility, low immunogenicity, easy reproducibility in preparation, and abundant sources. They can not only naturally encapsulate lipids, proteins, RNA, and various plant active small molecules, but also protect the active ingredients from degradation in the gastrointestinal tract, thereby enhancing their stability and bioavailability, and improving their tissue-targeted delivery capabilities [120]. It should be emphasized that the mechanistic evidence discussed in Section 4.2, Section 4.3, Section 4.4 and Section 4.5 is derived predominantly from studies of isolated or free plant-derived bioactive compounds rather than intact PELN preparations. These studies are therefore considered indirect evidence and are included only to provide mechanistic context for the potential contribution of PELN-associated cargo.

### 4.2. Polyphenols

Plant polyphenols (PP) are a class of naturally occurring secondary metabolites widely distributed in fruits, vegetables, tea, spices, and medicinal plants, primarily including flavonoids, phenolic acids, anthocyanins, tannins, lignans, and isoflavones [43]. Its biological activity mainly stems from the phenolic hydroxyl structure, which confers strong antioxidant capacity, attracting extensive attention in the fields of food, nutrition, and medicine.

Under normal physiological conditions, fat synthesis and breakdown maintain a dynamic equilibrium; when energy intake consistently exceeds the body’s requirements, the excess energy is stored in adipose tissue as triglycerides. However, once lipid metabolism becomes imbalanced, abnormal lipid accumulation can trigger various chronic metabolic diseases, posing serious threats to health. Recent studies have shown that PP may have a supportive role in improving MASLD, possibly through regulating related genes, antioxidant effects, and improving insulin resistance. Evidence concerning the effects of polyphenols is derived predominantly from studies of isolated compounds and therefore represents indirect evidence with respect to intact PELNs. Furthermore, the relevance of individual studies to MASLD differs. General evidence suggests that plant polyphenols can regulate lipid metabolism through pathways involving SREBP-1c, ACC, FASN, AMPK, and PPARα [132]. However, this evidence is primarily mechanistic rather than PELN-specific. Resveratrol (RSV) has been shown to activate sirtuin 1 (SIRT1) signaling and modify gut microbiota-derived metabolites in HFD-induced obesity models [133]. Secondly, research suggests that plant polyphenols can activate the Nrf2/HO-1 antioxidant signaling pathway, increase the activities of antioxidant enzymes such as SOD, glutathione peroxidase (GSH-Px), and catalase (CAT), and reduce oxidative damage indicators such as malondialdehyde (MDA), thereby alleviating liver cell damage caused by oxidative stress [134]. Epigallocatechin-3-gallate (EGCG) is the most abundant polyphenol isolated from green tea and possesses antioxidant properties and free radical scavenging capabilities. Its molecular structure is rich in multiple phenolic hydroxyl groups, which can efficiently quench reactive free radicals and maintain the integrity of the liver cell membrane structure and function. By contrast, isolated EGCG has been evaluated in an HFD-induced hepatic lipotoxicity model, in which it reduced mitochondrial ROS-associated injury [135].

However, most natural polyphenols have extremely low absorption rates in the digestive tract, resulting in a large portion of them accumulating in the colon region. This allows them to interact bidirectionally with the intestinal microecology and exert their physiological functions. Other studies describing the interactions between polyphenols and the gut microbiota provide additional indirect mechanistic support [136]. Curcumin, due to its excellent anti-inflammatory and antioxidant properties, is commonly used in various inflammatory diseases. However, it has extremely poor water solubility and a low absorption rate in the intestinal epithelium. It mainly needs to be metabolized by intestinal bacteria into small-molecule derivatives such as tetrahydrocurcumin (THC) before it can be absorbed into the bloodstream. Some researchers found through continuous oral administration of THC to mice for 8 weeks that THC significantly improved glucose metabolism and GLP-1 expression in diabetic db/db mice [137].

### 4.3. Alkaloids

Alkaloids (DNLA) constitute a large class of natural organic compounds mainly produced by plants, containing nitrogen and having significant biological activity. They are widely present in medicinal plants such as Coptis, Dendrobium, Sophora, and Schizonepeta. In MASLD, they are often accompanied by varying degrees of liver damage and oxidative stress. Studies have shown that alkaloids, as the active components of dendrobium, have a protective effect against liver damage induced by carbon tetrachloride. Evidence concerning alkaloids also originates from heterogeneous disease models. *Dendrobium nobile* alkaloids have been reported to alleviate CCl_4_-induced mitochondrial oxidative damage through Nrf2-associated antioxidant signaling [138], whereas leonurine(Leo) can regulate PI3K/Akt/Nrf2 and TLR4/NLRP3 signaling in acetaminophen-induced acute liver injury [139]. Because neither CCl_4_-nor acetaminophen-induced liver injury represents MASLD/MASH, these findings should be regarded as indirect mechanistic evidence relevant to oxidative and inflammatory pathways rather than direct MASLD evidence.

When MASLD exhibits severe lipid metabolism imbalance and increased fatty acid intake, DNLA can restore liver lipid homeostasis by regulating multiple aspects such as lipid synthesis, fatty acid oxidation, and cholesterol metabolism. Berberine (BBR), as a widely used plant extract in clinical practice, shows positive effects in various aspects, including glycolipid metabolism and antioxidant and anti-inflammatory activities. Studies have shown that BBR can activate AMPK through both direct and indirect mechanisms [140]. After AMPK is activated, it can directly phosphorylate and inhibit the activity of ACC, reducing the production of malonyl-CoA, thereby inhibiting fatty acid synthesis. Piperine exhibits various pharmacological effects in numerous in vitro and in vivo experiments. The experiments show that piperine intervention in mice can significantly downregulate the expression of adipogenic-related genes PPARγ and CD36, while upregulating hormone-sensitive lipase (HSL) and adipose triglyceride lipase (ATGL) [141]. These findings therefore represent MASLD -related compound evidence, although they do not constitute direct evidence for PELNs. In contrast, studies examining curcumin–piperine supplementation in obesity models [142] or the effects of berberine on gut microbiota [143] are considered indirect metabolic or gut–liver axis-related evidence.

### 4.4. Terpenoids

Terpenoids, also known as isoprenoids, are the most abundant and structurally diverse group of natural bioactive compounds in plants, composed of isoprene units. They are widely present in medicinal plants, aromatic plants, and fruits and vegetables, and exhibit various biological activities including anti-inflammatory, antioxidant, lipid metabolism regulation, antifibrotic, and immunomodulatory effects [144].

Ursolic acid (UA) is a pentacyclic triterpene primarily synthesized via the mevalonate (MVA) pathway. Despite its low solubility, poor bioavailability, and interactions with the gut microbiota upon oral administration, UA still exhibits strong pharmacological properties and biological activities [145]. Studies of terpenoids provide both direct MASLD-related and indirect mechanistic evidence. UA has been evaluated in experimental models of non-alcoholic fatty liver disease and hepatic steatosis, where it modulated LXRα/AMPK- and PPARα-associated pathways involved in lipid metabolism [146,147]. These findings are directly relevant to hepatic steatosis, although they remain evidence for the free compound rather than PELNs. NADPH oxidase 4 (NOX4) is abundantly present in hepatocytes and HSCs, and together with NLRP3, can induce various diseases. In contrast, the reported effects of UA on NOX4/NLRP3 signaling and liver fibrosis provide broader fibrotic and inflammatory mechanistic support but are not necessarily specific to MASLD [148]. Glycyrrhizic acid (GA) is extracted from the roots and rhizomes of the leguminous plant Glycyrrhiza, and is one of the main components responsible for its pharmacological activities. Similarly, the anti-inflammatory activity of free GA through HMGB1/TLR4/RAGE/NF-κB signaling should be considered indirect mechanistic evidence [149]. GA can also inhibit the activation of hepatic stellate cells by suppressing the TGF-β1/Smad signaling pathway and downregulating the expression of α-SMA, collagen I, and TIMP1 [150]. Oleanolic acid (OA) typically exists in free form or as a triterpene saponin aglycone, exhibiting strong lipid solubility but poor water solubility, resulting in relatively low oral bioavailability. OA can significantly alleviate hepatic steatosis by inhibiting de novo lipogenesis and activating the AMPK/PPARα pathway to enhance fatty acid β-oxidation [151]. Moreover, OA can improve gut microbiota composition and intestinal barrier integrity, highlighting the crucial role of the gut–liver axis in mediating its hepatoprotective effects.

### 4.5. Polysaccharides

Plant polysaccharides are natural macromolecular compounds formed by the linkage of multiple monosaccharides through glycosidic bonds. They are widely present in plant roots, stems, leaves, fruits, seeds, and fungi, and represent one of the key active components responsible for the biological functions of plants. Compared with polyphenols, alkaloids, and terpenoids, plant polysaccharides are characterized by their broad availability, excellent biocompatibility, low toxicity, and significant immunomodulatory effects, which have led to increasing attention in recent years. The disease relevance of evidence concerning plant polysaccharides also varies substantially.

Yellow tea polysaccharide (YTP) is a class of natural high-molecular-weight polysaccharide compounds extracted from yellow tea, typically composed of various monosaccharides such as glucose, galactose, arabinose, and rhamnose, and may form complexes with small amounts of proteins and phenolic compounds. YTP have been directly evaluated in an experimental MASLD model and were shown to regulate gut microbiota and bile acid metabolism [152]. Therefore, this study provides MASLD/NAFLD-related compound evidence. Its mechanism involves YTP altering the composition of colonic bile acids (BA), inhibiting ileal FXR and hepatic reabsorption of bile acids, and activating the liver FXR-SHP pathway. Rheum polysaccharides (LYP) have been shown to improve obesity-related metabolic disorders by potentially modulating the function of immune cells such as macrophages and lymphocytes. During the progression of obesity, polarization toward M1-like macrophages can exacerbate pro-inflammatory responses within adipose tissue, thereby inducing local and systemic insulin resistance. Experimental data indicate that LYP improves glucose and lipid homeostasis, increases energy expenditure, regulates pancreatic β-cell function, reduces M1 polarization and inflammatory response of adipose tissue macrophages (ATM) in mice, and prevents diet-induced obesity and metabolic syndrome [153]. Additionally, experiments have shown that LYP can be added as a nutritional supplement to enrich short-chain fatty acid-producing bacteria, sustain the integrity of intestinal mucosa and upregulate tight junction protein levels, which consequently strengthens the intestinal physical barrier capacity [154]. Because these studies primarily address obesity-associated metabolic dysfunction and intestinal homeostasis rather than hepatic steatosis, they are considered indirect evidence relevant to MASLD. Astragalus polysaccharides (APS) have been investigated in the context of NAFLD and may regulate PI3K/Akt and AMPK/ACC signaling, insulin sensitivity, autophagy, and mitochondrial function [155]. *Lycium barbarum* polysaccharides (LBP), one of the most important active components in the traditional Chinese medicine *Lycium barbarum* L., are primarily composed of monosaccharides such as arabinose, galactose, and glucose, and belong to water-soluble heteropolysaccharides. LBP, as a dietary intervention, significantly promotes the reconstruction of gut microbiota in HFD mice, particularly enriching *Allobaculum* and *Lactococcus*, while downregulating *Proteobacteria*, enhancing the production of short-chain fatty acids and GPCRs, and improving glucose homeostasis [156]. LBPs have been studied primarily in metabolic and gut microbiota-related models, including high-fat/high-fructose diet-induced metabolic abnormalities and glucose metabolism-related mechanisms [157]. Accordingly, the latter findings should be interpreted as indirect metabolic evidence rather than direct evidence for MASLD or PELNs.

**Figure 2:**
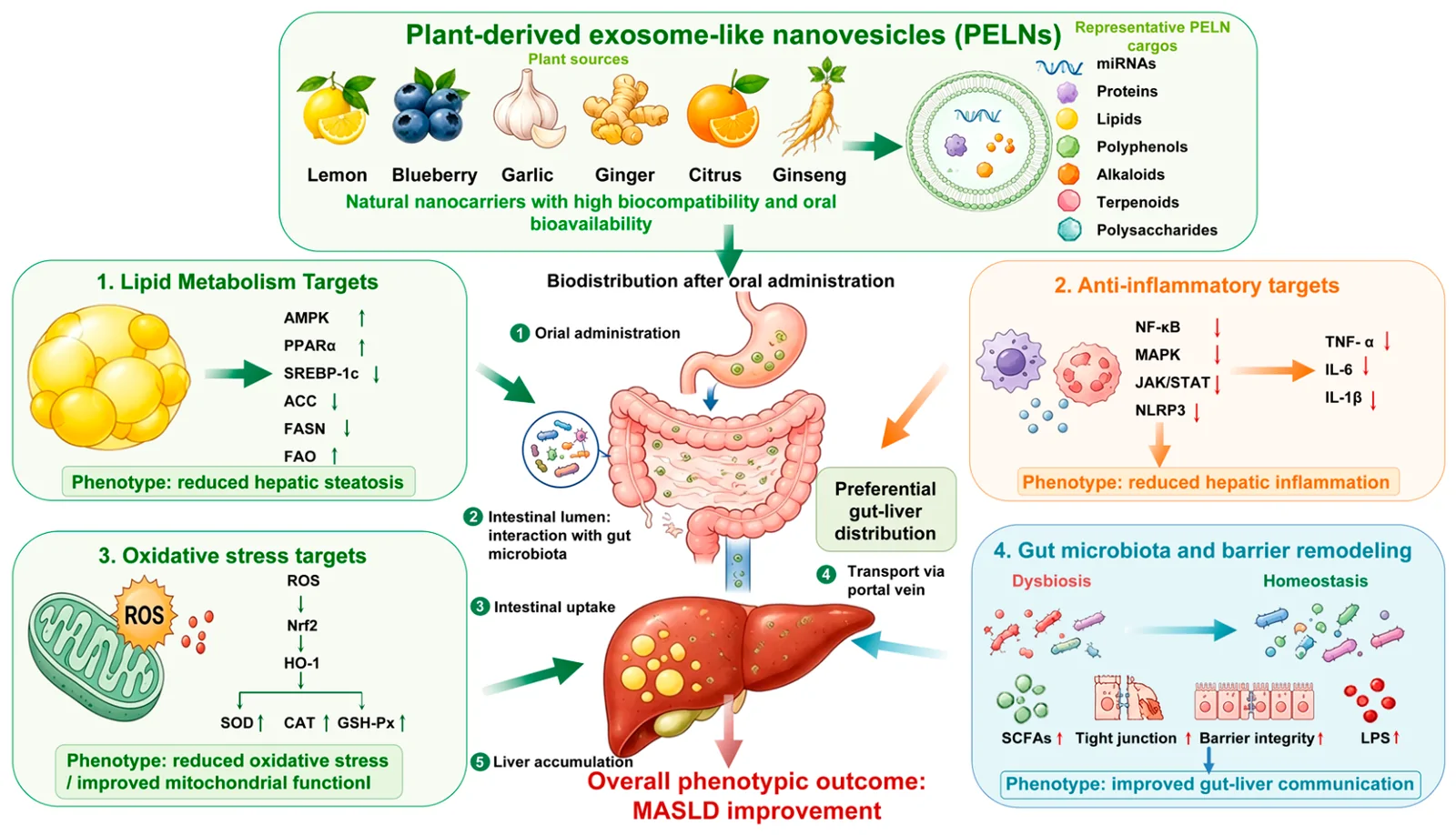
The mechanism of plant-derived exosome-like nanovesicles in treating MASLD.

Collectively, studies of isolated polyphenols, alkaloids, terpenoids, and polysaccharides provide indirect mechanistic evidence for signaling pathways that may potentially contribute to the biological activity of PELN-associated cargo. These pathways include the regulation of lipid metabolism, oxidative stress, inflammation, insulin signaling, and the gut microbiota. However, the activity of an isolated compound cannot be assumed to represent the biological activity of the corresponding PELN preparation. Direct validation using characterized PELNs is therefore required to establish cargo-specific mechanisms. The mechanism of PELNs in treating MASLD is summarized in Figure 2.

## 5. PELNs from Different Sources

### 5.1. Fruit-Derived PELNs

In contrast to Section 4, which focuses primarily on mechanistic evidence derived from isolated plant bioactive compounds, this section summarizes studies that directly investigated plant-derived vesicular preparations. However, not all experimental models represent MASLD or MASH. Accordingly, studies that directly assessed hepatic steatosis- or MASLD-related liver pathology are distinguished from studies conducted in obesity, diabetes, insulin resistance, gut dysbiosis, or other related models, which are considered indirect evidence for MASLD.

Fruit-derived PELNs are rich in lipids, proteins, polyphenols, flavonoids, vitamins, and other bioactive compounds. Blueberries are abundant in anthocyanins, flavonoids, polyphenols, and various antioxidant components; the extracellular vesicles derived from them can carry these bioactive substances and exert biological functions through intercellular communication. Blueberry-derived exosome-like nanovesicles (BELNs) have been shown to regulate antioxidant gene expression via Nrf2 activation, reduce intracellular ROS levels, enhance mitochondrial membrane potential, and inhibit apoptosis by upregulating the expression of anti-apoptotic protein Bcl-2 and HO-1 [130]. However, because the specific BELN component responsible for this response has not been conclusively established, the observed Nrf2 modulation should not be interpreted as evidence that intact BELNs directly target Nrf2. In addition, this research model can serve as direct preclinical evidence for the treatment of MASLD with PELNs. Another set of experimental data shows that high-dose citrus-derived exosome-like nanovesicles (CELNs) significantly increased the abundance of most short-chain fatty acids (SCFAs) in HFD mice, reduced the relative abundance of the pathogenic bacterium *Prevotellamassilia timonensis*, and simultaneously enhanced the levels of beneficial bacteria (*Acetatifactor muris* and *Phocaeicola sartorii*) [158]. Tangerine peel-derived exosome-Like nanovesicles (TNVs) derived from orange peels can enhance the expression of genes related to fatty acid oxidation (FAO), while simultaneously inhibiting the expression of genes related to fat production and glycolysis in the liver [159]. TNVs treatment was also associated with enhanced hepatic FXR-SHP signaling, increased bile acid excretion, and reduced hepatic cholesterol accumulation. Importantly, TNVs simultaneously altered the gut microbiota and bile acid metabolism. Therefore, the observed FXR-SHP response may represent a downstream consequence of changes in bile acid composition and/or PELN-associated cargo activity rather than direct molecular activation of FXR by the vesicles themselves. The specific vesicular component responsible for this signaling effect remains to be identified. Lemon-derived nanovesicles (LNVs) can significantly reduce the generation of ROS in liver cells, thereby activating the Nrf2/HO-1 signaling pathway, upregulating the expression of Nrf2 protein in the nucleus, and thereby improving glucose tolerance impairment and lipid metabolism disorders in rats induced by a high-fat diet [160]. However, the available evidence does not establish that LNVs directly regulate Nrf2 at the molecular level. Because hepatic steatosis was directly evaluated, this study is considered direct hepatic steatosis-related evidence despite the underlying diabetic model. Similarly, pomegranate-derived exosome-like nanovesicles (PENs) were investigated in an HFD-induced NAFLD model and alleviated hepatic mitochondrial dysfunction and oxidative stress through SIRT3/SOD2-associated mechanisms, providing direct MASLD-related evidence [161].

### 5.2. Vegetable-Derived PELNs

Garlic itself possesses strong antibacterial activity and can reduce oxidative stress. Therefore, garlic-derived exosome-like nanovesicles (GaELNs) may also exert similar effects. In an experiment, GaELNs were orally administered to mice fed a high-fat diet, revealing that they were preferentially taken up by microglia, suppressed brain inflammation in HFD mice, and alleviated obesity via the gut–brain axis [162]. Because the principal outcomes involved obesity, neuroinflammation, glucose tolerance, and insulin sensitivity rather than hepatic steatosis, this study is considered indirect metabolic evidence relevant to MASLD. The formation of the GaELN/BASP1 complex suppresses c-Myc-mediated STING expression, thereby downregulating the production of inflammatory cytokines IFN-γ and TNF-α. This prevents activation of the aryl hydrocarbon receptor (AHR) pathway, ultimately delaying the progression of obesity. Garlic-derived nanovesicles can also modulate the expression of PFKFB3, a key glycolytic enzyme, through macrophage-hepatocyte interaction, inhibiting inflammation and alleviating liver dysfunction in high-fat diet-fed mice [163]. Although this study provides liver-related mechanistic evidence, it is classified conservatively as indirect MASLD-relevant evidence because direct assessment of hepatic steatosis- or MASH-related pathology was not its principal endpoint. Functional components in kidney beans play a positive role in regulating intestinal microbiota balance, reducing fat, combating obesity, and lowering blood glucose. Animal studies have shown that intervention with kidney bean-derived exosome-like nanovesicles (KBELNs) effectively alleviates obesity and promotes the production of SCFAs [164]. Meanwhile, 16S rRNA gene sequencing analysis revealed that oral administration of KBELNs significantly enhanced both α-diversity and β-diversity of the gut microbiota, thereby modulating the intestinal microbial community structure. Specifically, KBELNs selectively enriched beneficial bacterial genera (such as *Bifidobacterium*) while significantly suppressing the abundance of potential pathogenic bacteria (such as *Shigella*), thus ameliorating obesity phenotypes induced by a high-fat, high-protein diet. These findings therefore represent indirect gut–metabolic evidence relevant to MASLD rather than direct MASLD evidence. Ginger-derived nanoparticles (GDNP) can restore the downregulated Foxa2 expression caused by a high-fat diet by inhibiting Akt-1-mediated phosphorylation [165]. Meanwhile, GDNP can reconstruct intestinal epithelial homeostasis and effectively prevent high-fat diet-induced obesity and insulin resistance. Moreover, the experiment found that oral administration of GDNP not only significantly extended the lifespan of mice but also markedly alleviated skin inflammatory responses. Accordingly, these findings are considered indirect evidence relevant to insulin resistance, a major component of MASLD pathophysiology. Based on these characteristics, studies have proposed the development of recombinant curcumin derivative nanovesicles (Rec-tNVs) to improve curcumin encapsulation efficiency and further validate their potential role in obesity regulation. Functionally, Rec-tNVs can specifically activate the TRPV1 channel and induce intracellular calcium elevation, thereby promoting systemic lipid metabolism, enhancing lipolysis, suppressing lipogenesis, driving the conversion of white adipocytes into thermogenic beige adipocytes, and ultimately triggering adipocyte apoptosis [166]. These findings are therefore considered indirect metabolic evidence rather than direct MASLD evidence. Exosome-like nanoparticles derived from mung bean sprouts (MELNs) have been shown in anti-diabetic research to enhance the transcriptional activity of Nrf2 by activating the PI3K/Akt/GSK-3β signaling pathway, thereby promoting its nuclear translocation. Such regulatory events elevate the levels of pivotal antioxidant enzymes including HO-1 and SOD, which effectively mitigates oxidative stress and ameliorates insulin resistance within hepatocytes [167]. However, because the active vesicular cargo or membrane component responsible for these signaling changes was not separately established, direct targeting of this pathway by intact MELNs cannot be concluded. Because this model primarily represents diabetes, these findings provide indirect mechanistic evidence relevant to MASLD. In the MASLD mouse model, intervention with broccoli-derived exosome-like nanoparticles (BDENs) significantly reduced the ALT/AST ratio and increased the HDL/LDL ratio, demonstrating that BDENs effectively alleviate liver pathological damage [168]. Additionally, it can maintain the intestinal barrier by improving the gut–liver axis, regulate bile acid metabolism in the gut, and restore the gut microbiota.

### 5.3. Herb-Derived PELNs

Honeysuckle-derived exosome-like nanovesicles (HNVs) are nanoscale vesicular preparations obtained from dried honeysuckle flowers. Oral administration of HNVs enriched with bioactive compounds significantly suppresses the increase in lipid vacuoles, lipid droplet accumulation, and collagen fibrosis in the livers of MASLD mice [169]. HNVs exert their effects through dual intestinal-liver interventions. On one hand, HNVs repair the intestinal barrier, reduce serum LPS levels, and alleviate liver inflammation. On the other hand, they improve microbial metabolism, elevate secondary bile acid taurochenodeoxycholic acid (TCDCA) levels, inhibit the intestinal-liver axis FXR-FGF15-fibroblast growth factor receptor 4 (FGFR4) pathway, and lower cholesterol levels in both the liver and serum. Because these signaling changes occurred together with substantial remodeling of the gut microbiota and bile acid profile, the FXR-related effects are more appropriately interpreted as downstream gut–liver responses rather than evidence of direct molecular inhibition of FXR signaling by HNVs. Tea-derived exosome-like nanoparticles (TELNs) exhibit significant antioxidant activity, effectively alleviating oxidative stress in the body, enhancing hepatocyte survival rates, and reducing ROS accumulation. Studies have shown that TELNs significantly ameliorate oleic acid-induced lipid metabolic disorders and liver cell damage. Further molecular mechanism analysis revealed that TELNs downregulate the expression of miR-21-5p, miR-17-3p, and miR-107, thereby upregulating the expression of their target genes PPARα, cholesterol 7α-hydroxylase (CYP7A1), and CPT-1A, promoting lipid metabolism and improving hepatic lipid metabolic disorders [170]. Because this is an in vitro hepatocyte lipid accumulation model rather than a complete MASLD model, these results are considered MASLD-relevant mechanistic evidence but not direct in vivo MASLD evidence. In contrast, fermented black tea-derived exosome-like nanoparticles were evaluated in an experimental MASLD model and improved hepatic lipid accumulation and associated metabolic abnormalities [171], providing direct preclinical evidence relevant to MASLD. Modern research has shown that ginseng is rich in various active components such as ginsenosides, polysaccharides, and volatile oils, which possess multiple potential biological functions. Ginseng-derived nanovesicles (GNVs), enriched with bioactive substances like ginsenosides, can cross biological barriers and exert significant biological activities. Studies have demonstrated that oral administration of GNVs significantly alleviates liver injury and fibrosis in both MASLD and alcoholic liver disease (ALD) mouse models [172]. Its mechanism for treating MASLD primarily involves restoring the expression of intestinal tight junction proteins and improving intestinal barrier function, thereby significantly reducing intestinal permeability. Further studies have revealed that GNVs promote extracellular matrix degradation and inhibit liver fibrosis by modulating the gut–liver axis and the TIMP2 signaling pathway. Their effects are considered direct MASLD-related evidence. In contrast, findings obtained in the alcohol-associated liver disease arm should be regarded as supportive hepatoprotective evidence rather than direct evidence for MASLD. *Centella asiatica*-derived nanovesicles (ADNVs) are rich in multiple miRNAs that can target cancer. Further studies have shown that ADNVs primarily affect metabolic pathways such as amino acid metabolism and lipid biosynthesis [173]. After treatment with ADNVs, the proportion of HepG2 cells in the G0/G1 phase significantly increased, while the proportion in the G2/M phase significantly decreased, indicating that ADNVs can arrest the cell cycle of HepG2 cells, inhibit DNA replication, and ultimately lead to cell death. Additionally, ADNVs elevated intracellular ROS levels and reduced mitochondrial membrane potential, thereby inducing early apoptosis in cells. At the same time, ADNVs significantly decreased the aspartate content in HepG2 cells. These results suggest that ADNVs exert anti-hepatocarcinoma effects by modulating metabolic pathways and related metabolites, highlighting a close association between these metabolites and the mechanisms of liver cancer therapy.

The findings and features discussed above are summarized in Table 2.

## 6. Current Issues and Challenges

Although PELNs show promising preclinical potential for MASLD-related intervention due to their wide availability, favorable biocompatibility, relatively low immunogenicity, and potential delivery advantages, current research remains largely confined to cellular and experimental animal studies, and veterinary translation still faces numerous challenges. Another important limitation is the heterogeneity of disease models used to evaluate PELNs. Although several studies have directly investigated hepatic steatosis or MASLD, a substantial proportion of the available evidence originates from models of obesity, diabetes, insulin resistance, gut dysbiosis, or other metabolically related conditions. These models reproduce individual components of MASLD pathophysiology but do not fully recapitulate the hepatic phenotype of MASLD or MASH. Therefore, therapeutic effects observed in these models should not be directly extrapolated to MASLD. Future studies should prioritize well-characterized MASLD/MASH models and incorporate standardized liver-specific endpoints, including hepatic lipid content, histological steatosis, lobular inflammation, hepatocyte ballooning, and fibrosis-related outcomes.

Another major challenge is the lack of standardized characterization and quality assessment of PELN preparations. Differences in plant source, tissue preprocessing, isolation procedures, purification strategies, storage conditions, and analytical methods can result in substantial variation in particle size distribution, particle concentration, purity, yield, cargo composition, and biological activity [100,115,116,121]. In addition, plant-derived preparations may contain co-isolated membrane fragments, soluble proteins, polysaccharides, free metabolites, and residual isolation reagents, which may independently contribute to the observed biological effects. Consequently, therapeutic outcomes obtained using crude or partially purified preparations cannot always be attributed exclusively to the vesicular fraction. Future studies should therefore report particle size and concentration, morphology, preparation purity, vesicle yield, and representative lipid, protein, RNA, and bioactive cargo profiles in a standardized manner. Batch-to-batch variability should also be carefully evaluated, because differences in plant materials, processing procedures, and storage conditions may alter vesicle yield and cargo composition and thereby affect biological activity.

The biodistribution and liver-targeting properties of PELNs also remain insufficiently defined. Although selected animal studies have reported intestinal uptake and subsequent distribution of orally administered PELNs to extraintestinal tissues, including the liver, these findings cannot be generalized to all PELN preparations. Tissue distribution may vary according to plant source, particle size, membrane and cargo composition, administered dose, route of administration, and preparation purity. Moreover, qualitative fluorescence or tracer signals detected in hepatic tissue should not automatically be interpreted as evidence of quantitative liver targeting. Comprehensive pharmacokinetic studies evaluating absorption, tissue exposure, residence time, metabolism, and clearance remain scarce. Future studies should therefore establish dose–exposure relationships and determine whether the reported hepatic effects are associated with measurable delivery of intact vesicles or their functional cargos to the liver. For oral administration in ruminants, the rumen represents a distinct physiological environment that may influence the fate of PELNs before they reach the intestine. Encouragingly, the existing relevant evidence indicates that the vesicular nanoparticles derived from plants can maintain their structural integrity under gastrointestinal conditions, suggesting that the rumen environment of ruminants does not necessarily lead to significant damage to the vesicles [126,174]. This stability may support the feasibility of oral PELN delivery in ruminant livestock. Nevertheless, current evidence remains limited to specific plant-derived vesicle preparations and experimental conditions, and it remains unclear whether these findings can be generalized across PELNs from different plant sources. Further studies should therefore evaluate vesicle integrity, cargo retention, biological activity, and subsequent intestinal uptake under both simulated rumen conditions and in vivo ruminant models.

Safety assessment represents another important limitation. Current evidence is derived largely from short-term preclinical observations, and the absence of obvious adverse effects under a specific experimental condition should not be interpreted as evidence of established long-term safety. Repeated administration may influence immune recognition, inflammatory responses, tissue accumulation, and clearance. Moreover, potential adverse effects may arise not only from the vesicles themselves but also from co-isolated plant components or residual processing reagents. Future studies should therefore systematically evaluate repeated-dose toxicity, hematological and biochemical parameters, major-organ histopathology, immunogenicity, tissue accumulation, clearance, and potential off-target effects. These assessments should ultimately be extended to relevant livestock species using dosing regimens that reflect the intended route and duration of veterinary administration [20].

Manufacturing reproducibility and quality control are also essential for future veterinary translation. Current PELN production lacks unified standards for plant raw-material selection, preprocessing, isolation, purification, concentration, formulation, storage, and transportation [115,116]. Scaling up production may further increase variation in vesicle recovery, purity, cargo composition, and biological activity. Therefore, critical quality attributes should be defined for each production batch, including vesicle identity, particle size distribution and concentration, purity, yield, membrane integrity, representative cargo composition, residual contaminants, biological potency, and storage stability. Batch-to-batch consistency should be evaluated using predefined acceptance criteria to ensure that preparations produced at different times remain comparable. For engineered PELNs, additional parameters such as cargo-loading efficiency, surface modification, structural integrity, and residual processing reagents should also be monitored. Establishing reproducible manufacturing procedures and standardized quality-control frameworks will be essential before practical veterinary application can be considered.

## 7. Summary and Outlook

Current evidence suggests that PELNs represent a promising preclinical approach for the investigation of MASLD-related interventions. Selected PELN preparations have shown beneficial effects on hepatic steatosis, oxidative stress, inflammation, metabolic dysfunction, and gut–liver axis disturbances in cellular and experimental animal models. However, the overall evidence base remains limited and heterogeneous. Only a subset of studies has directly evaluated MASLD or hepatic steatosis, whereas many others have been conducted in obesity, diabetes, insulin resistance, gut dysbiosis, or other metabolically related models. In addition, several proposed mechanisms are supported primarily by studies of isolated plant bioactive compounds rather than intact PELNs. Therefore, the currently reported biological effects should be interpreted as preclinical evidence and should not be considered sufficient to establish the therapeutic effectiveness or practical applicability of PELNs for metabolic liver disease.

Substantial experimental and translational work is still required before the practical veterinary applicability of PELNs can be established. Major priorities include standardized characterization of vesicle preparations, improved control of purity and non-vesicular contaminants, clarification of active cargo composition, and greater consistency in isolation, purification, and dosing procedures. Quantitative biodistribution and pharmacokinetic studies are also needed to determine intestinal uptake, tissue exposure, hepatic delivery, persistence, metabolism, and clearance, rather than assuming general liver-targeting properties across different PELNs. In parallel, repeated-dose toxicity, immunogenicity, tissue accumulation, off-target effects, and long-term safety require systematic evaluation. Future development will additionally depend on scalable and reproducible manufacturing, control of batch-to-batch variability, predefined quality attributes and release criteria, appropriate storage conditions, and the establishment of relevant regulatory and quality-control frameworks. Finally, validation in economically important livestock species under physiologically and production-relevant conditions will be essential. Until these requirements are addressed, PELNs should be regarded as experimental nanovesicular candidates with promising preclinical potential rather than established veterinary therapeutic strategies for MASLD-related metabolic liver disorders.

## Figures and Tables

**Table 1:** Comparison of commonly used methods for the isolation and purification of plant-derived exosome-like nanovesicles.

Isolation/Purification Method	Principle	Relative Yield/Recovery	Purity	Major Advantages	Major Limitations	Scalability	Application Suitability	Ref.
Differential centrifugation + ultracentrifugation (UC)	Sequential low- to high-speed centrifugation removes large debris and enriches vesicles according to sedimentation behavior	Moderate–high	Moderate	Widely used; relatively simple workflow; no additional chemical precipitant required; allows direct enrichment of vesicular particles	Requires expensive ultracentrifugation equipment; time-consuming; possible vesicle aggregation or structural damage; protein aggregates, membrane fragments and particles with similar sedimentation properties may co-precipitate	Low–moderate	Routine laboratory PELN isolation; suitable for morphological characterization and biological assays when very high purity is not essential	[14,100,115,121]
Density-gradient centrifugation	Vesicles are separated according to buoyant density using sucrose or other density-gradient media	Low–moderate	High	Provides better separation from soluble proteins, cell-wall fragments and other non-vesicular particles; improves preparation purity and population resolution	Labor-intensive and time-consuming; requires ultracentrifugation; lower throughput and recovery may occur because of multiple separation steps; residual gradient medium may require removal	Low	Particularly suitable for high-purity PELN preparations, mechanistic studies, cargo analysis, proteomics and studies requiring reduced non-vesicular contamination	[14,100,116]
Size-exclusion chromatography (SEC)	Separates particles according to hydrodynamic size; larger vesicles pass through the porous matrix earlier than smaller soluble molecules	Moderate	Moderate–high	Mild separation conditions; preserves vesicle membrane integrity and biological activity; effectively reduces contamination by soluble proteins; highly reproducible	Limited sample-loading capacity; dilute samples often require pre-concentration; multiple fractions must be collected; relatively inefficient for very large sample volumes	Moderate	Suitable for functional studies, downstream molecular characterization and applications in which preservation of intact biologically active vesicles is important	[100,115,121]
Polyethylene glycol (PEG) precipitation	PEG decreases vesicle solubility and promotes precipitation, followed by low- or moderate-speed centrifugation	High	Low	Simple operation; inexpensive; no ultracentrifuge required; suitable for processing relatively large sample volumes; high apparent particle recovery	Poor selectivity; co-precipitates soluble proteins, polysaccharides, lipoprotein-like particles and other plant-derived components; residual PEG may interfere with proteomic, nucleic-acid and functional analyses	Moderate–high	Useful for rapid enrichment, preliminary screening and pre-concentration; additional purification is recommended before detailed mechanistic, omics or therapeutic studies	[13,100,121]
Commercial kit-based isolation	Proprietary precipitation, membrane-affinity or column-based reagents are used to enrich vesicle-containing fractions	Moderate–high	Low–moderate/variable	Rapid; convenient; simple standardized workflow; requires little specialized equipment; suitable for small sample numbers	Relatively expensive; proprietary composition may limit methodological transparency; co-isolation of non-vesicular material can occur; batch- and kit-dependent recovery and purity; difficult to compare across studies	Low–moderate	Suitable for exploratory studies and small-scale rapid isolation; less suitable for mechanistic studies, quantitative comparisons or large-scale production unless purity is independently validated	[100,116,121]

**Table 2:** Application of PELNs from different plant sources in rodent obesity models.

PELNs Source	PELNs Preparation/Characterization	Animals (Sex; *n*)	Experimental Model and Disease Induction	Relevance to MASLD/MASH	Effect After PELNs Treatment	Dose and Route	Liver Histology
Blueberry [130]	Vesicle-enriched preparation; PEG-based isolation; nanoparticle morphology/size and molecular cargo characterized	C57BL/6 mice; sex NR; *n* = 5–8 for reported endpoints	HFD for 8 weeks before BELN intervention	Direct MASLD/NAFLD model	Decreased ROS levels, reduced AST and ALT levels, upregulated expression of anti-apoptotic proteins (Bcl-2, Bax, HO-1), suppressed expression of fat synthesis-related genes (FASN, ACC)	Mass-based: 25, 50, or 100 mg/kg per dose, twice daily for 4 weeks	Yes; H&E and Oil Red O
Cirtus [158]	CELNs isolated and physicochemically characterized; exact purification grade not clearly reported	Male C57BL/6J mice, 8 weeks old; *n* NR	Short-term LFD/HFD model: 3-day LFD followed by 7-day HFD	Indirect MASLD-relevant metabolic/gut dysbiosis model	Increased abundance of SCFAs, decreased abundance of pathogenic bacteria (*Prerotellamassilia timonensis*), increased abundance of beneficial bacteria (*Acetatifactor muris* and *Phocaeicola sartorii*)	Particle-based: 2 × 10^12^, 1 × 10^13^, or 5 × 10^13^ particles/kg/day for 10 days	No
Lemon [160]	Industrial lemon nanovesicles; physicochemical and biochemical characterization performed; purity not defined as a highly purified vesicle fraction	Male Wistar rats; total *n* = 10	HFD-induced metabolic alterations for 10 weeks before intervention	Indirect MASLD-relevant HFD metabolic model	Decreased ROS levels, increased nuclear Nrf2 and HO-1 expression, reduced serum TG and LDL-C levels	Mass-based: 1.2 mg/kg/day for 4 weeks	Partial; hepatic Nrf2 immunohistochemical evaluation performed
Pomegranate [161]	Exosome-like nanovesicles isolated and physicochemically characterized	Mice; sex NR; *n* NR	HFD-induced NAFLD for 9 weeks	Direct MASLD/NAFLD model	Serum levels of ALT, TG, and TC decreased, GSH/GSSG levels in the liver returned to normal, expression of antioxidant proteins (SIRT3, SOD2) increased	Mass-based: 25 or 50 mg/kg/day for 9 weeks	Yes; Oil Red O
Garlic [162,163]	Differential centrifugation + 150,000× *g* ultracentrifugation + sucrose-gradient purification; TEM characterization;	Male C57BL/6 mice, 10–12 weeks old; *n* = 5 for several reported endpoints	Long-term HFD-induced obesity/insulin resistance; HFD for >1 year before treatment	Indirect MASLD-relevant obesity/insulin-resistance model	Reduced levels of inflammatory factors (IFN-γ and TNF-α), improved glucose tolerance and insulin sensitivity, changes associated with attenuation of cGAS/STING/IDO1/AHR-related inflammatory signaling, decreased expression of the glycolytic enzyme PFKFB3	Particle-based: 1 × 10^10^ particles/day for 6 weeks;	Yes; liver H&E
Kidney bean [164]	Exosome-like nanovesicles isolated and characterized	Rats; sex and *n* NR	12-week HFD-induced obesity	Indirect MASLD-relevant obesity/gut dysbiosis model	Increased SCFA production, beneficial bacteria (*Bifidobacterium*) increased, harmful bacteria (*Escherichia-Shigella*) decreased	Mass-based: 10.21, 20.42, or 40.84 g/kg/day for 12 weeks	NR
Ginger [165]	Differential centrifugation followed by density/sucrose fractionation; NTA/TEM characterization	Male C57BL/6 mice, 6–8 weeks old; *n* = 10/group in the principal experiment	Long-term HFD-induced obesity and insulin resistance	Indirect MASLD-relevant obesity/insulin-resistance model	Foxa2 expression returned to normal, prevented HFD-induced insulin resistance, extended mouse lifespan, suppressed skin inflammation	Particle concentration: 6 × 10^8^ particles/mL in drinking water	No
Turmeric [166]	Reconstructed nanovesicles, rather than unmodified native PELNs; extensive physicochemical characterization performed	C57BL/6 mice; sex/*n* NR	VHFD/HFD-induced obesity models	Indirect MASLD-relevant obesity model	Weight loss, reduction in adipose tissue volume, modulation of adipogenesis-related transcription factor expression (upregulation of CEBPα, downregulation of PPARG1 and PPARG2)	Particle-based: 4.9 × 10^11^ particles/kg per administration, twice daily; 6.1 × 10^11^ particles/kg per administration, twice daily	No
Mung bean sprout [167]	Differential centrifugation + PEG isolation; TEM, PAGE, agarose gel electrophoresis and TLC characterization; vesicle-enriched/partially purified preparation	Male C57BL/6 mice, 8 weeks old; *n* NR	HFD for 4 weeks followed by STZ (100 mg/kg, i.p.) to induce T2DM	Indirect MASLD-relevant T2DM model	Decreased serum AST, ALT, TG, and TC levels, increased PI3K/Akt-associated signaling responses, increased production of antioxidant enzymes HO-1 and SOD	Mass-based: 40 mg/kg/day for 5 weeks	Yes; liver H&E
Broccoli [168]	Differential centrifugation from fresh broccoli; particle characterization and miRNA profiling performed	Male C57BL/6J mice, 6 weeks old; *n* = 24 total, 8/group	HFD for 8 weeks before BDEN treatment	Direct MASLD mode	Weight loss, reduced ALT/AST ratio, restoration of gut microbiota balance, serum LPS levels	Mass-based: 100 mg/kg/day for 4 weeks	Yes; hepatic histopathology and Oil Red O
Honeysuckle [169]	Exosome-like nanovesicles prepared from dried *Lonicera japonica*; morphology and particle-size characterization; bioactive metabolites characterized	C57BL/6 mice; sex/*n* NR in accessible report	HFD for 10 weeks before intervention	Direct MASLD/MAFLD model	Restoration of intestinal barrier integrity, reduced serum LPS levels, inhibition of hepatitis, reshaping of gut microbiota	Mass-based: 25, 50, or 100 mg/kg per dose, twice daily for 6 weeks	Yes; H&E, Oil Red O and Sirius Red
Tea leaf [171]	TELNs before/after *Aspergillus cristatus* fermentation; particle size/concentration and biochemical/metabolomic characterization	40 male C57BL/6J mice, 8 weeks old	HFD-induced NAFLD; 2-week HFD induction before intervention	Direct MASLD/NAFLD model	Weight loss, significant reductions in TC, TG, ALT, AST, IL-6, and TNF-α levels, decreased lipid droplet accumulation	Volume-based: 0.2 mL/day for 4 weeks	Yes; Oil Red O
Ginseng [172]	Ginseng-derived exosome-like nanovesicles isolated and characterized; vesicular fraction used experimentally	Mice; sex/*n* NR in accessible report	FFC diet-induced MASLD with steatosis/inflammation/fibrosis	Direct MASLD/MASH-like model	Oxidative stress suppression, gut microbiota remodeling, inhibition of HSC activation, reduction in hepatic lipid accumulation and oxidative damage	Mass-based: 0.5 or 1 mg/kg/day for 5 weeks; ALD model: 1 mg/kg/day for 2 weeks	Yes; Hepatic pathology evaluated

Note: NR indicates information that was not reported or could not be clearly determined from the original study.

## Data Availability

No new data were created or analyzed in this study. Data sharing is not applicable to this article.
